# Protective effects of *Stevia rebaudiana* extracts on beta cells in lipotoxic conditions

**DOI:** 10.1007/s00592-021-01793-9

**Published:** 2021-09-09

**Authors:** Marco Bugliani, Silvia Tavarini, Francesca Grano, Silvia Tondi, Serena Lacerenza, Laura Giusti, Maurizio Ronci, Anna Maidecchi, Piero Marchetti, Marta Tesi, Luciana G. Angelini

**Affiliations:** 1 Department of Clinical and Experimental Medicine, University of Pisa, Pisa, Italy; 2 Department of Agriculture, Food and Environment, University of Pisa, Pisa, Italy; 3 Aboca SpA Società Agricola, Sansepolcro, Tuscany, Italy; 4 Department of Pharmacy, University of Pisa, Pisa, Italy; 5 School of Pharmacy, University of Camerino, Camerino, Italy; 6 Department of Pharmacy and Centre for Advanced Studies and Technologies (CAST), University “G. D’Annunzio” of Chieti-Pescara, Chieti, Italy; 7 Centro Interdipartimentale NUTRAFOOD, University of Pisa, Pisa, Italy

**Keywords:** *Stevia rebaudiana*, Steviol glycosides, Beta cells, Lipotoxicity, Proteomics

## Abstract

**Aims:**

*Stevia rebaudiana* Bertoni leaf extracts have gained increasing attention for their potential protection against type 2 diabetes. In this study, we have evaluated the possible beneficial effects of *Stevia rebaudiana* leaf extracts on beta-cells exposed to lipotoxicity and explored some of the possible mechanisms involved.

**Methods:**

Extracts, deriving from six different chemotypes (ST1 to ST6), were characterized in terms of steviol glycosides, total phenols, flavonoids, and antioxidant activity. INS-1E beta cells and human pancreatic islets were incubated 24 h with 0.5 mM palmitate with or without varying concentrations of extracts. Beta-cell/islet cell features were analyzed by MTT assay, activated caspase 3/7 measurement, and/or nucleosome quantification. In addition, the proteome of INS-1E cells was assessed by bi-dimensional electrophoresis (2-DE).

**Results:**

The extracts differed in terms of antioxidant activity and stevioside content. As expected, 24 h exposure to palmitate resulted in a significant decrease of INS-1E cell metabolic activity, which was counteracted by all the Stevia extracts at 200 μg/ml. However, varying stevioside only concentrations were not able to protect palmitate-exposed cells. ST3 extract was also tested with human islets, showing an anti-apoptotic effect. Proteome analysis showed several changes in INS-1E beta-cells exposed to ST3, mainly at the endoplasmic reticulum and mitochondrial levels.

**Conclusions:**

*Stevia rebaudiana* leaf extracts have beneficial effects on beta cells exposed to lipotoxicity; this effect does not seem to be mediated by stevioside alone (suggesting a major role of the leaf phytocomplex as a whole) and might be due to actions on the endoplasmic reticulum and the mitochondrion.

## Introduction

Type 2 diabetes (T2D) is the most common form of diabetes [1], and it is due to a complex interaction of genetic and acquired factors leading to beta-cell failure [2, 3]. Among the acquired factors that are known to be associated with T2D, an important role is played by the deleterious effects of increased concentrations of glucose (glucotoxicity) and certain fatty acids (lipotoxicity) [4, 5]. Hence, many studies have tried to address how to protect beta cells from metabolic insults [5–9]. In this regard, *Stevia rebaudiana* Bertoni (Stevia), a native plant to the northeast of Paraguay, mainly known for its sweetener properties, has recently been shown to have beneficial effects in several models of disease and cellular dysfunction, including diabetes and beta cells [10–19]. Stevia leaf extracts represent, in fact, a unique Natural Complex Substance, thanks to the presence of steviol glycosides, phenolic acids, flavonoids, alkaloids, water-soluble chlorophylls, xanthophylls, hydroxycinnamic acids, minerals, and vitamins [20]. The majority of these phytochemicals have a wide variety of biological activities, which makes Stevia a plant source with potential benefits for human health [21]. Accordingly, Stevia’s “antidiabetic” effects seem due to the bioactive compounds present in the medium-polar and aqueous extracts [22–24]. These effects are generally thought to be due to steviol glycosides, the molecules that confer sweet flavor to Stevia extracts, although also the non-sweetener fraction might display insulinotropic effects [22]. More than 30 steviol glycosides have been detected in Stevia leaves [23], but the most abundant are stevioside, rebaudioside A and rebaudioside C. In literature, there are studies regarding the action of stevioside in stimulating insulin secretion, both in-vitro and in-vivo [24–32]. No data are currently available on the possible protective effects of Stevia extracts on pancreatic beta cells upon prolonged exposure to certain free fatty acids, also in relation to Stevia secondary metabolites – mainly steviol glycosides, polyphenols, and flavonoids—and their related antioxidant and ROS-scavenging activities. Therefore, the aim of this study was to evaluate the effects of leaf extracts from some *Stevia rebaudiana* Bertoni chemotypes, characterized by different phytochemical profiles, on beta-cells exposed to lipotoxic conditions. First, the leaf extracts were characterized in terms of total phenols and flavonoids, free radical-scavenging activity, total antioxidant capacity, steviol glycoside profile, and content. Then, the protective effects of Stevia leaf extracts were evaluated in INS-1E beta cells and human islets exposed to lipotoxicity. Finally, by proteomic analysis, we assessed the changes induced by Stevia in INS-1E cells.

## Materials and methods

### Chemicals

Ethanol and ferrous sulfate were purchased from Carlo Erba SpA (Milan, Italy). Water and acetonitrile were obtained from JT Baker (Phillipsburg, NJ, USA). Pure stevioside, rebaudioside A, rebaudioside B, rebaudioside C, rebaudioside D, dulcoside A and steviolbioside (97% pure) were purchased from ChromaDex Inc. (Irvine, CA, USA). Gallic acid monohydrate (3,4,5-trihydroxy benzoic acid), TPTZ (2,4,6-tri(2-pyridyl)-s-triazine), Trizma acetate, Folin–Ciocalteu reagent, sodium carbonate, and ferric chloride were all obtained from Sigma-Aldrich Chemical Co. (Milan, Italy). All chemicals used in the present study, including solvents, were of analytical grade.

### Plant material

Six stevia chemotypes (ST1 and ST2 from the germplasm collection of the Department of Agriculture, Food and Environment—DAFE; ST3, ST4, ST5, and ST6 from ABOCA germplasm collection) were used in this study. They were organically cultivated in open field conditions at the Experimental Centre of DAFE, located at San Piero a Grado, in Pisa coastal plain (Pisa, Central Italy 43° 40’ N of latitude and 10° 20’ E of longitude) following the Guidelines on Good Agricultural and Collection Practice (GACP) of the European Medicines Agency.

The leaves of each chemotype were collected at the end of the vegetative growth, before the start of flowering, when the content in bioactive compounds, especially steviol glycosides, was maximum. The leaves were dried in a ventilated oven at 40 °C until constant weight, ground to a fine powder in a laboratory mill, and kept in sealed bags at room temperature until the subsequent extraction procedure.

### Preparation of crude extracts

The dried leaves of the six Stevia chemotypes were extracted using hydroalcoholic solvent (ethanol 50% v/v) for 6 h at 40 °C, by means of a Naviglio extractor, programmed with 51 cycles of 7 min. The plant material/solvent ratio was 1/10. Each cycle included a static phase of five min and a dynamic phase of two min, with 6 pumps min^−1^. The working pressure was 6–8 bar. The crude extract was centrifuged, dried under reduced pressure, and finally lyophilized to give an olive-green powder.

### Steviol glycoside analysis

Steviol glycoside (SVgly) determination was performed according to Zimmermann et al. [33] and Tavarini et al. [20]. Operating HPLC conditions and chromatogram acquisition were based on the procedure described by Tavarini et al. [34]. SVgly composition and quantification were performed using a standard curve of an authentic standard mixture (0.05–0.5 g L^−1^), containing stevioside, rebaudioside A, rebaudioside B, rebaudioside C, rebaudioside D, dulcoside A, and steviolbioside.

### Total phenolic content

Total phenols were determined by the Folin-Ciocalteu method, according to Dewanto et al. [35] with some modifications [20]. In order to calculate the standard curve, gallic acid was used as an external standard. The results were expressed as mg gallic acid equivalent (GAE) g^−1^ of lyophilized extract.

### Total flavonoids determination

Total flavonoids were spectrophotometrically determined using the method described by Barros et al. [36], based on the aluminum chloride assay. The measurements were taken at a wavelength of 510 nm using a UV–visible spectrophotometer (Varian Cary 1E, Palo Alto, CA U.S.A.). Catechin was used as an external standard to calculate the standard curve, and the results were expressed as mg of catechin equivalents (CE) g^−1^ of lyophilized extract.

### Ferric reducing antioxidant power (FRAP) assay

Total antioxidant activity was determined based on the method by Benzie and Strain [37] with slight modifications [20]. Trolox was used as standard, and the final values were expressed as mmol Trolox equivalent (TE) g^−1^ of lyophilized extract.

### INS-1E beta cells and human islets

INS-1E cells (a kind gift of prof. Claes B. Wollheim) were cultured in RPMI 1640 completed with 10% fetal bovine serum (Thermo Fisher Scientific, Paisley, UK), 10 mM hepes (Sigma-Aldrich, Saint Louis, MO, USA), 100 mg/ml penicillin–streptomycin (Sigma-Aldrich), 1 mM sodium pyruvate (Thermo Fisher Scientific), 50 μM 2-mercaptoethanol (Thermo Fisher Scientific) at 37 °C in 5% CO_2_ atmosphere. Human islets were isolated from 9 non-diabetic organ donors (age: 72 ± 12 y, gender: 5 M/4F; BMI: 26.3 ± 4.7 kg m^−2^) by enzymatic digestion followed by density gradient purification, and cultured in M199 medium complete with 10% bovine serum and antibiotics, as previously described [38, 39], until the day of the experiment.

To evaluate the effects of the different extracts of stevia on beta cells, INS-1E cells were cultured 24 h in RPMI 1640 complete with 1% fatty acid-free BSA and 1% FBS with or without 0.5 mM palmitate [39], in presence or not of increasing concentrations (from 10 μg/ml to 1000 μg/ml) of extracts. In other experiments, human islets were cultured for 24 h in presence of 0.5 mM palmitate [4, 8, 40] with or without 80 μg/ml of a stevia extract in M199 medium completed with antibiotics and 1% fatty acid-free BSA.

### MTT assay

Cell viability was monitored indirectly through the MTT assay [41, 42]. This assay estimates cell metabolic efficiency by exploiting the ability of the mitochondrial enzyme NAD(P)H-dependent cellular oxidoreductase enzymes to reduce the 3-(4,5-dimethylthiazol-2-yl)-2,5-diphenyltetrazolium bromide salt (MTT) to its insoluble derivative formazan, which has a purple color. Briefly, a solution containing 5 mg/ml of tetrazolium bromide salt (Sigma-Aldrich) dissolved in PBS was added to the cells exposed to each condition to get a final concentration of 0.5 mg/ml. Then, the plate was kept in an incubator at 37 °C and 5% CO_2_ for 3 h. In the end, the supernatant was aspirated, and the formed salt dissolved in DMSO. After 20 min of shaking, the plate was read in a plate reader at 570 nm with a baseline at 650 nm. The viability was calculated as a percentage of absorbance with respect to untreated cells.

### Apoptosis evaluation

Human islet cell apoptosis was evaluated by caspase activation and an ELISA method. Caspase 3/7 activity was measured by Caspase-Glo® 3/7 Assay (Promega Corporation, Madison, WI, USA) following the manufacturer protocol. Briefly, 15 hand-picked islets were cultured in lipotoxic conditions (3–5 experimental points for each condition) and then a working solution containing a tetrapeptide linked to aminoluciferin was added to the cultured cells. Following caspase cleavage of the substrate, aminoluciferin is released and, in the presence of luciferase and ATP, results in the luciferase reaction and production of light which, in turn, can be detected by a luminometer. In additional sets of experiments, apoptosis was also evaluated by Cell Death Detection Elisa^Plus^ kit (Roche Diagnostics, Mannheim, Germany) as per manufacturer indications.

### Proteome analysis

INS-1E cultured 24 h in presence of 200 µg/ml of ST3 were washed twice with PBS (37 °C), suspended in the rehydration solution (7 M urea, 2 M thiourea, 4% CHAPS, 60 mM DTT, 0.002% bromophenol blue), and incubated for 1 h at room temperature (RT). Thereafter, the samples were centrifuged at 13,000 g for 10 min at RT to remove undissolved material. In order to remove all the interfering substances, the samples have been treated with 2-D Clean-Up Kit (GE Health Care Europe; Uppsala, Sweden) using manufacture instructions. Protein concentration of the resulting supernatant was determined using the Bio-Rad RC/DC-protein assay (Bio-Rad, Hercules, CA, USA) with BSA as a standard [43]. 2-DE analysis was performed on beta-cell protein extracts. Briefly, 200 μg of proteins were filled up to 450 μL in rehydration solution supplemented with 0.8% v/v IPG Buffer, pH 3–10 NL (GE Healthcare), and 1% (v/v) Pharmalyte 3–10. Immobilien Dry-Strips (GE Health Care Europe; Uppsala, Sweden), 18 cm, nonlinear gradient pH 3–10 were rehydrated overnight in the sample and then transferred to the Ettan IPGphor II apparatus (GE Health Care). Isoelectrofocusing (IEF) was performed at 16 °C and the proteins were focused for up to 70000Vh. The second dimension (Sodium Dodecyl Sulphate–Polyacrylamide Gel Electrophoresis; SDS-PAGE) was carried out by transferring the proteins to 12.5% polyacrylamide gel, running at 16 mA/gel and 12 °C for about 16 h. The gels were stained with Ruthenium II tris (bathophenanthroline disulfonate) tetrasodium salt (SunaTech Inc.; Suzhou, P. R. China) (RuBP) as previously described [43]. Gel images were acquired by “Image Quant LAS4010” and the analysis of 2-DE images was performed using the Same Spot (v4.1, TotalLab; Newcastle Upon Tyne, UK) software. A comparison between cells treated with and without ST3 was performed. The spot volume ratios between the two different conditions were calculated using the average spot normalized volume of the three biological replicates. The software included statistical analysis calculations. The protein spots of interest with *p* < 0.05, were cut out from the gel, trypsin digested, and identified by Nano-liquid chromatography-electrospray ionization tandem mass spectrometry (nano-LC–ESI–MS/MS) analysis as previously described [44]. DataAnalysis v. 4.2 was used to process the raw data and generate the peak list to be submitted to the database search through BioTools 3.2 exploiting the free version of MASCOT search engine against Uniprot/Swiss-Prot non-redundant database version 2014–11. Rattus norvegicus taxonomy was specified for database searching [43]. Protein–protein interaction networks were analyzed using string software (string-db.org/). Confidence view was assigned a score of 0.4, indicating medium confidence.

### Statistical analysis

Data from phytochemical screening were subjected to analysis of variance (ANOVA) using CoStat version 6.2 (CoHort Software, Monterey, CA, USA). One-way completely randomized ANOVA was carried out to estimate the effect of biotype, followed by least significant difference (LSD) posthoc test. *p* < 0.05 was considered to indicate statistical significance. Prior to ANOVA, inhibition percentage data were subjected to an arcsine transformation. Cellular data were presented as mean ± SEM. To represent the results of this part of the study, data from control experiments were expressed as 100% at each given condition. The effects of the perturbators were then expressed for each experiment as variation from the respective control values. Differences between the groups were analyzed by ANOVA followed by Bonferroni correction or Dunnet T3 test as appropriate. A *p*-value < 0.05 was considered statistically significant.

## Results

### Phytochemical evaluation of the stevia leaf extracts

The tested Stevia chemotypes showed significant differences in terms of phytochemical composition (Table 1). ST2 was characterized by the highest content of phenols and flavonoids, while ST5 and ST6 showed the lowest content. Accordingly, ST2 reached a significantly higher total antioxidant activity (FRAP), and both ST5 and ST6 extracts exhibited the lowest antioxidant values (Table 1). Regarding the total steviol glycoside content, both ST1 and ST3 showed the highest amount, while ST4 extract was characterized by the lowest content (Table 1). The profile of the main steviol glycosides is reported in Table 2. The most abundant of them were, as expected, stevioside, rebaudioside A, and rebaudioside C. The content of the most known compound, stevioside, was highest in ST3 extract and lowest in ST2 and ST4 extracts.

**Table 1:** Total phenols, total flavonoids, total antioxidant activity (measured by FRAP assay), and total SVgly content in the leaf extracts of the six stevia chemotypes

	Total Phenols (mg GAE g^−1^ LE)	Total Flavonoids(mg CE g^−1^ LE)	FRAP (mmol TE g^−1^ LE)	Total SVglys (g 100 g^−1^ LE)
ST1	154.99 ± 6.66^a^	128.08 ± 0.81^a^	1.51 ± 0.08^a^	40.57 ± 0.13^e^
ST2	175.95 ± 2.40^a^	150.24 ± 4.57^a^	2.31 ± 0.10^a^	35.58 ± 0.09^f^
ST3	117.72 ± 2.88^a^	105.34 ± 5.80^a^	1.25 ± 0.06^a^	40.88 ± 0.06^e^
ST4	93.39 ± 4.17^a^	39.46 ± 0.94^c^	0.94 ± 0.3∙10^−1a^	28.04 ± 0.59^a^
ST5	63.01 ± 2.48^b^	31.04 ± 0.09^d^	0.68 ± 0.02^b^	33.25 ± 0.35^a^
ST6	64.39 ± 0.50^b^	28.32 ± 0.97^b^	0.59 ± 0.01^b^	34.67 ± 0.27^f^
One-way ANOVA	*p* < 0.001	*p* < 0.001	*p* < 0.001	*p* < 0.001

Results are the means (*n* = 3) ± SE. A one-way ANOVA was used to evaluate the effect of chemotype. The LSD post-hoc test was also used:

^a^Significantly different versus all the other values within the column;

^b^Significantly different versus ST1-ST4 within the column;

^c^Significantly different versus ST1-ST3 and ST6 within the column;

^d^Significantly different versus ST1-ST3 within the column;

^e^Significantly different versus ST2 and ST4-ST6 within the column;

^f^Significantly different versus ST1 and ST3-ST5 within the column

*FRAP* ferric reducing antioxidant power, *SVgly* steviol glycoside, *GAE* gallic acid equivalent, *LE* lyophilized extract, *CE* catechin equivalents, *TE* Trolox equivalent, *LSD* least significant difference

**Table 2:** Concentration of the main SVglys identified in leaf extracts of the six stevia chemotypes

	Stbio (g 100 g^−1^ LE)	Dulc A (g 100 ^−1^ LE)	Stev (g 100 g^−1^ LE)	Reb C (g 100 g^−1^ LE)	Reb A (g 100 g^−1^ LE)
ST1	1.94 ± 0.01^a^	4.59 ± 0.01^a^	12.81 ± 0.08^a^	10.40 ± 0.05^d^	10.83 ± 0.09^a^
ST2	1.18 ± 0.03^b^	6.43 ± 0.05^a^	9.64 ± 0.05^c^	10.46 ± 0.03^d^	7.87 ± 0.02^a^
ST3	1.14 ± 0.01^b^	4.16 ± 0.01^a^	22.41 ± 0.05^a^	3.03 ± 0.01^e^	10.14 ± 0.02^a^
ST4	0.83 ± 0.01^a^	2.61 ± 0.08^a^	9.28 ± 0.52^c^	6.09 ± 0.25^a^	9.23 ± 0.10^f^
ST5	1.34 ± 0.05^a^	1.66 ± 0.03^a^	17.43 ± 0.28^a^	3.39 ± 0.19^e^	9.43 ± 0.08^f^
ST6	5.33 ± 0.09^a^	3.02 ± 0.02^a^	15.31 ± 0.23^a^	2.07 ± 0.10^a^	8.94 ± 0.08^a^
One-way ANOVA	*p* < 0.001	*p* < 0.001	*p* < 0.001	*p* < 0.001	*p* < 0.001

Results are the means (*n* = 3) ± SE. A one-way ANOVA was used to evaluate the effect of the biotype. The LSD post-hoc test was also used:

^a^Significantly different versus all the other values within the column;

^b^Significantly different versus ST1 and ST4-ST6 within the column;

^c^Significantly different versus ST1, ST3 and ST5-ST6 within the column;

^d^Significantly different versus ST3-ST6 within the column;

^e^Significantly different versus ST1-ST2, ST4 and ST6 within the column;

^f^Significantly different versus ST1-ST3 and ST6 within the column

*SVglys* steviol glycosides, *Stbio* steviolbioside, *Dulc A* dulcoside A, *Stev* stevioside, *Reb C* rebaudioside C, *Reb A* rebaudioside A, *LE* lyophilized extract, *LSD* least significant difference

Taken together, these results underline the influence of the chemotype (i.e. a chemically distinct biotype with differences in the composition of the secondary metabolites) in defining the phytochemical composition of stevia leaf extracts.

### Direct effects of stevia extracts and stevioside on INS-1E and human islet cells

**Fig. 1:**
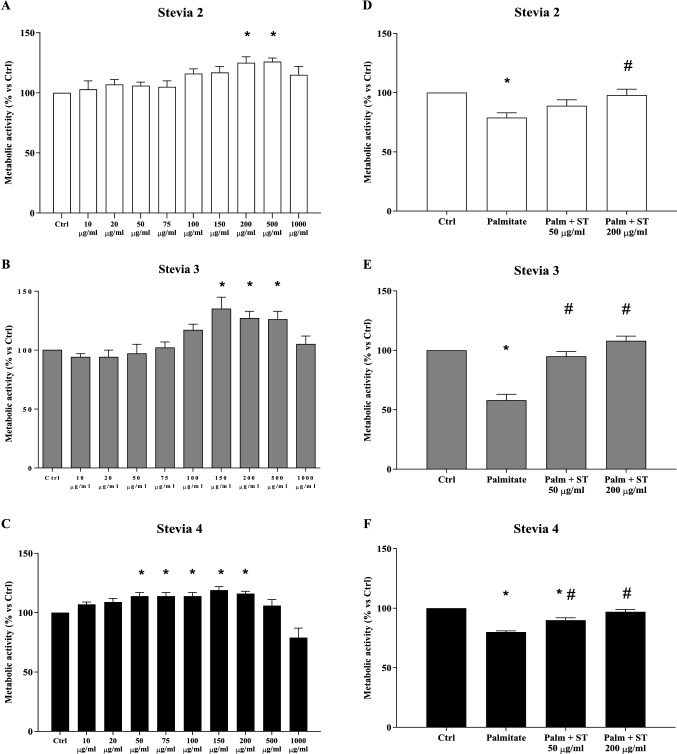
Metabolic activity of INS-1E cells exposed to increasing concentrations of the different stevia extracts (Panels A, B and C). *p < 0.05 vs Ctrl by ANOVA followed by Dunnet’s T3 test. Effects of different stevia extracts on INS-1E cells cultured 24 h in 0.5 mM palmitate (Panels D, E, and F). *p < 0.05 versus Ctrl; #*p* < 0.05 versus Palmitate by ANOVA followed by Dunnet’s T3 test. Data refer to 3 separate experiments, each consisting of 5 replicates

Taking into account the phytochemical composition of the extracts, we decided to test the effects of extracts with different antioxidant activity and stevioside content (namely ST2, ST3, and ST4) on INS-1E cells indirectly measuring cell viability with the use of MTT. After 24 h incubation, no sign of cytotoxicity was observed for concentrations of the extracts ranging from 10 μg/ml to 1000 μg/ml. Cell metabolic activity increased with 200 and 500 μg/ml for ST2, 150, 200, and 500 μg/ml for ST3 and from 50 to 200 μg/ml for ST4 (Fig. 1, panels A-C). Based on these results, we decided to use a concentration in the lower range (50 μg/ml) and one in the higher range (200 μg/ml) to assess the effects of the extracts against lipotoxicity. Palmitate exposure induced a reduction of beta-cell metabolic activity ranging, approximately, 20–40% compared to control samples. A beneficial effect was observed for ST3 and ST4 at 50 μg/ml and for all the 3 extracts at 200 μg/ml (Fig. 1, panels D–F).

**Fig. 2:**
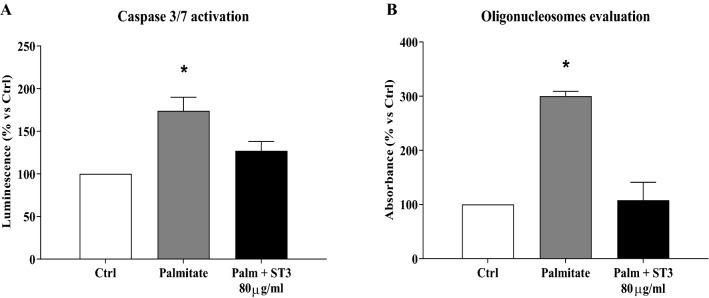
Caspase 3/7 activation in human islets exposed to 0.5 mM palmitate with (black bar) or without (grey bar) the presence of 80 μg/ml of stevia 3 (ST3) extract. *p < 0.05 versus Ctrl by ANOVA followed by Dunnet’s T3 test (panel A). Islet cell death measured by oligonucleosome evaluation in human islets exposed to 0.5 mM palmitate with (black bar) or without (grey bar) the presence of 80 μg/ml of ST3 extract. *p < 0.05 versus all groups by ANOVA followed by Dunnet’s T3 test (panel B). Data refer to 3–5 experiments, each consisting of 3 replicates

Since ST3 was able to prevent the detrimental action of palmitate both at 50 and 200 μg/ml, we assessed whether a similar positive effect could be reproduced also on human islets exposed to the lipotoxic condition in the presence of an intermediate concentration of the extract (80 μg/ml). By a luminescent technique, it was observed that palmitate treatment induced significant activation of caspase 3/7, which was prevented by the presence of ST3 (Fig. 2, panel A). In addition, there was a threefold increase of apoptosis in palmitate-treated islets, which was prevented by ST3 co-treatment (Fig. 2, panel B).

**Fig. 3:**
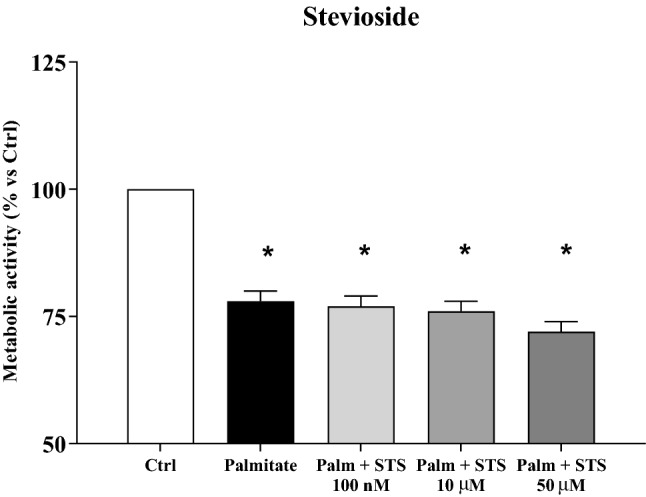
Effect of increasing concentrations of stevioside (STS) on the metabolic activity of INS-1E cells exposed for 24 h to 0.5 mM palmitate, measured by MTT assay. *p < 0.05 versus Ctrl by ANOVA followed by Dunnet’s T3 test. Data refer to 3 separate experiments, each consisting of 5 replicates

In order to assess whether the observed positive effects of stevia extracts could be mediated, at least in part, by stevioside, we evaluated beta-cell viability on INS-1E cells exposed to palmitate, with or without increasing concentrations of this compound. As shown in Fig. 3, palmitate-induced a marked reduction of metabolic activity, and the presence of stevioside at the tested concentrations did not prevent this detrimental effect.

### Proteomic studies

**Fig. 4:**
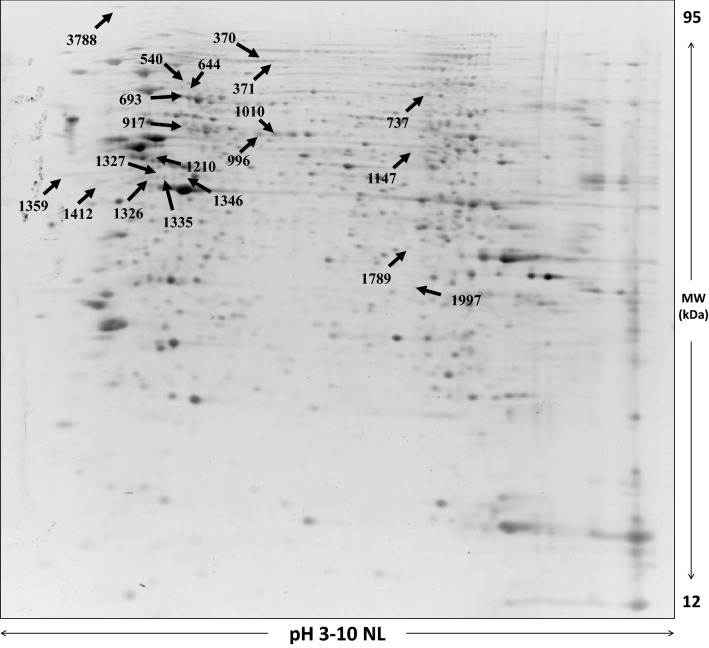
Representative 2D gel map of INS-1E beta cells. The number indicates the proteins identified by nano LC-ESI MS/MS and reported in Table 3

**Fig. 5:**
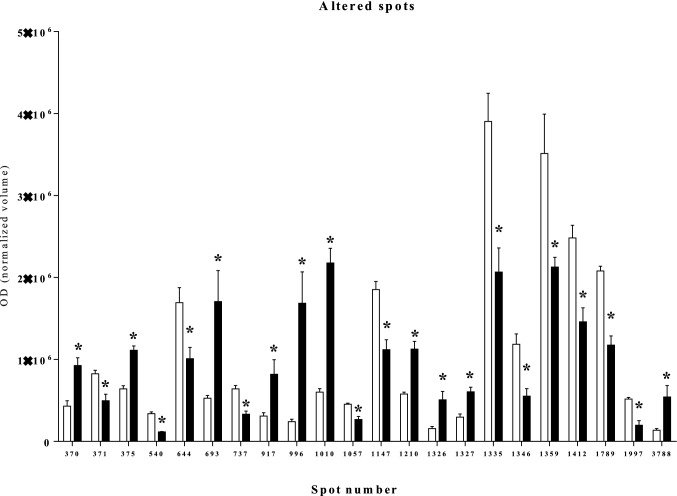
Histograms of the normalized OD density volumes of the protein spots found significantly different in stevia-treated INS-1E beta cells (black bars) with respect to untreated cells (white bars). *p < 0.05 versus untreated cells by ANOVA

To get some insights on the mechanisms involved in the effects of stevia extracts, we analyzed the proteome modification induced in INS-1E by 24 h exposure to 200 μg/ml of ST3. A representative image of beta-cell protein extract is shown in Fig. 4. After computational comparison of images obtained by treated and untreated beta-cell protein extracts, 22 spots resulted to be differentially expressed (fold change > 1.7). Of them, 10 were increased and 12 decreased after treatment with ST3. Figure 5 reports bar charts of the spots found differentially expressed and Table 3 shows the list of the identified proteins (more than one identification was displayed for the spots 3788, 693, 1210, 1147, and 1997), MS/MS results, protein expression ratio, *p*-values and subcellular localization. Interestingly, most of them are associated with the endoplasmic reticulum and the mitochondrion (Table 3).

**Table 3:** List of proteins differentially expressed identified by nano LC-MS/MS spectrometry. Coverage indicates the percentage of the protein sequence covered by identified peptides. Isoelectric point (pI) represents the pH value at which the overall charge of the protein is zero (neutral) and consequently it does not migrate in an electric field

Spot #	ID	Protein name	Gene name	Coverage (%)	Number peptide	MW (dalton)	pI	Anova (*p*-value)	Ratio (Stevia/Control)	Subcellular Localization
370	D3ZAN3	Alpha glucosidase 2 alpha neutral subunit	Ganab	11	9	90,573	5.77	0.012	2.2	ER
371	D3ZAN3	Alpha glucosidase 2 alpha neutral subunit	Ganab	11	9	90,573	5.77	0.044	0.58	ER
540	A0A0G2JVH4	MICOS complex subunit MIC60	Immt	33	26	86,230	5.62	1.94E-04	0.34	MITO
644	P06761	Endoplasmic reticulum chaperone BiP	Hspa5	36	21	72,347	5.01	0.04	0.58	ER
693	P63018	Heat shock cognate 71 kDa protein	Hspa8	51	34	70,871	5.37	0.007	3.2	NUCLEUS
693	F1M953	Stress-70 protein, mitochondrial	Hspa9	24	13	73,745	5.87	0.007	3.2	MITO
737	Q9ER34	Aconitate hydratase, mitochondrial	Aco2	10	7	85,433	7.15	0.007	0.53	MITO
917	P63039	60 kDa heat shock protein, mitochondrial	Hspd1	54	34	60,956	5.35	0.026	2.6	MITO
996	P11598	Protein disulfide-isomerase A3	Pdia3	58	35	56,623	6.78	0.001	7	ER
1010	P11598	Protein disulfide-isomerase A3	Pdia3	49	29	56,623	6.78	3.21E-04	3.6	ER
1147	Q6AXV4	Sorting and assembly machinery component 50 homolog	Samm50	26	12	51,960	6.34	0.014	0.58	MITO
1147	P70541	Translation initiation factor eIF-2B subunit gamma	Eif2b3	27	12	50,436	6.54	0.014	0.58	CYTO
1147	P11980	Pyruvate kinase	Pkm	17	9	57,818	6.63	0.014	0.58	CYTO
1210	Q63081	Protein disulfide-isomerase A6	Pdia6	38	16	48,173	4.95	0.002	1.9	ER
1210	P10719	ATP synthase subunit beta, mitochondrial	Atp5f1b	28	12	56,354	4.95	0.002	1.9	MITO
1326	Q5VLR5	BWK4	Erp44	17	7	46,878	5.08	0.011	3.2	ER
1327	Q5VLR5	BWK4	Erp44	5	2	46,878	5.08	0.012	2	ER
1335	Q5VLR5	BWK4	Erp44	46	19	46,878	5.08	0.017	0.53	ER
1346	D3ZZC1	RCG43947	Txndc5	24	8	46,353	5.58	0.015	0.47	ER
1346	Q6P3V8	Eukaryotic translation initiation factor 4A1	Eif4a1	13	5	46,154	5.32	0.015	0.47	CYTO
1359	G3V6S3	Calumenin	Calu	21	7	37,064	4.49	0.037	0.58	ER
1412	D3ZUB0	Reticulocalbin 1	Rcn1	26	11	38,090	4.59	0.014	0.58	ER
1789	Q99MZ8	LIM and SH3 domain protein 1	Lasp1	65	18	29,970	6.61	0.005	0.55	CYTO
1997	D3ZL85	Cytochrome c heme lyase	Hccs	18	4	31,130	6.25	0.025	0.38	MITO
1997	P04797	Glyceraldehyde-3-phosphate dehydrogenase	Gapdh	13	3	35,828	8.14	0.025	0.38	CYTO
3788	P11598	Protein disulfide-isomerase A3	Pdia3	43	23	56,623	6.78	0.009	4	ER
3788	P18418	Calreticulin	Calr	25	10	47,996	4.33	0.009	4	ER

**Fig. 6:**
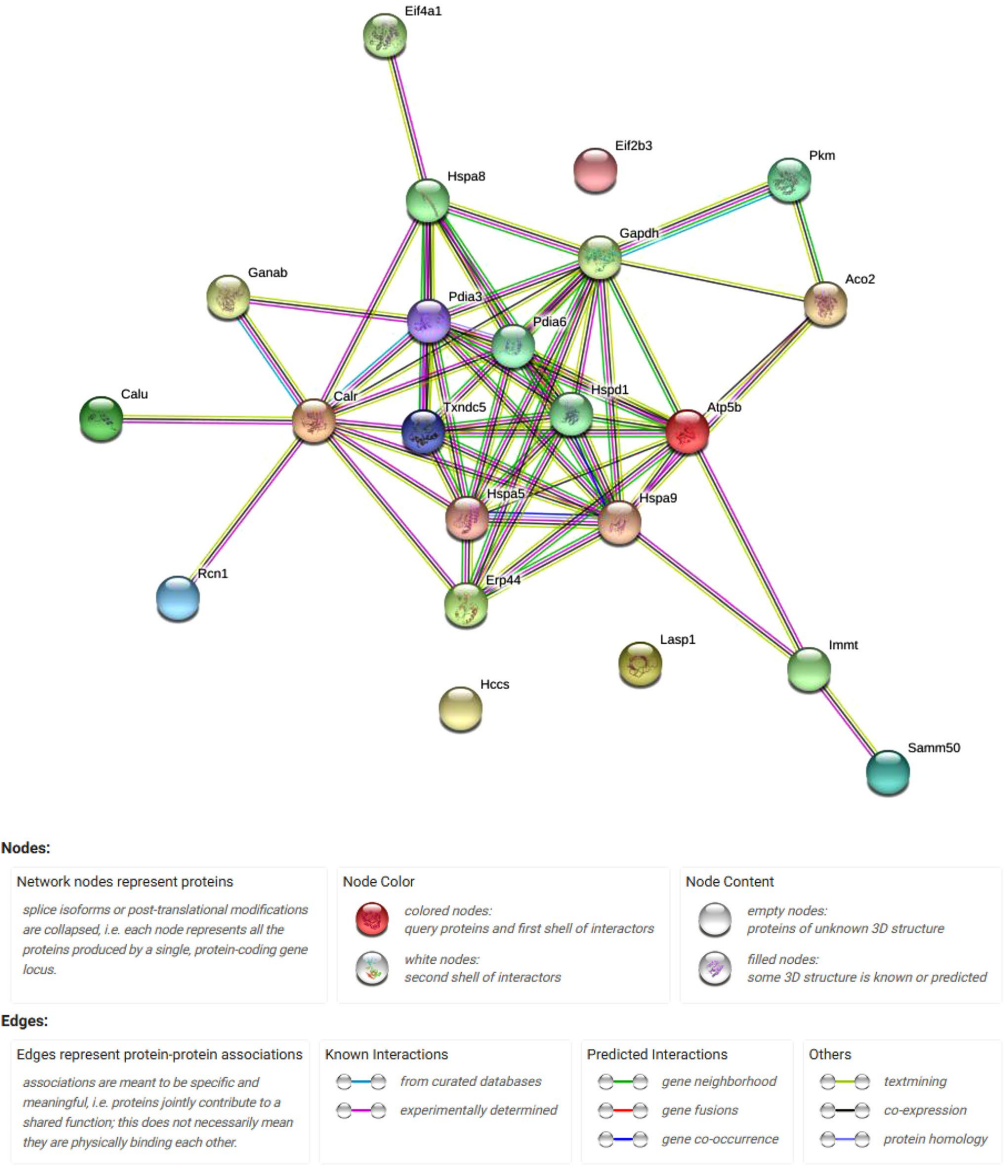
Predicted protein–protein interactions map of differentially expressed proteins after ST3 treatment of INS-1E beta cells. The interaction map was generated using STRING online tool with default parameters. Network nodes represent proteins. Edges represent protein–protein associations. Confidence view was assigned a score of 0.4, indicating medium confidence. The two top biological processes were protein folding and response to stress with a false discovery rate (FDR) value of 11.6 e^−4^ and 4.0 e^−4^_,_ respectively. Network stats: number of nodes, 22; number of edges, 59; average node degree, 5.36; average local clustering coefficient, 0.558; expected number of edges, 6; Protein–protein interaction (PPI) enrichment *p*-value, < 10e^−15^

To investigate the relationship between the differentially expressed proteins, protein–protein interactions were analyzed by STRING software. The STRING network analysis (Fig. 6) described pathways associated with protein folding, metabolism of proteins, and response to endoplasmic reticulum stress.

## Discussion

This study describes the phytochemical characteristics of several different Stevia leaf extracts and reports on the direct protective actions of some of such extracts on beta-cells exposed to lipotoxicity.

To date, most studies on the action of Stevia on beta cells have addressed insulin secretory features [10, 13, 17–19, 22, 25, 27–32]. In our study, we focused on parameters markers of cell survival/death, which were evaluated in a beta-cell line (INS-1E) and isolated human islets. These two models are often combined to provide basic information and potential translational insights [39, 45]. In addition, although human islets are heterogeneous due to the presence of different endocrine cells, beta cells are the most represented ones and evidence exists to indicate that they are the islet cells most susceptible to palmitate-induced damage [46, 47].

The six chemotypes that we studied (named ST1 to ST6) showed apparent differences in their chemical composition and properties. These include the levels of phenols, flavonoids and steviol glycosides, and antioxidant activity. Such differences can be due to genetic characteristics, as well as cultivation factors [48, 49]. In particular, we observed that ST2 was characterized by the highest content of phenols and flavonoids as well as high antioxidant activity, ST1 and ST3 showed the highest steviol glycoside concentration, ST4 exhibited the lowest quantity of glycosides, whereas ST5 and ST6 had the lowest antioxidant properties. Despite these differences, extracts with different phytochemical composition (ST2, ST3, and ST4 in the present study) demonstrated similar effects on the viability of INS-1E beta cells, leading to an improvement of the metabolic activity of the cells at similar concentrations. This is in line and implements previous evidence generated in-vitro and in-vivo with fewer and less characterized Stevia extracts [10, 13, 17–19, 22, 25, 27–32].

Interestingly, the ST2, ST3, and ST4 chemotypes were able to similarly protect INS-1E beta cells from the lipotoxic action due to prolonged exposure to the saturated fatty acid, palmitate. Indeed, palmitate reduced cell viability by 20–40%, confirming previous work with this model [39, 50, 51]. However, the presence of ST2, ST3, and ST4 prevented palmitate-induced beta-cell damage in INS-1E beta cells. To assess if specific Stevia extract components were responsible for this protective effect, INS-1E, treated with palmitate, were exposed to stevioside only. This main stevia component has been shown to ameliorate insulin secretion in INS-1E cells exposed to palmitate or high glucose concentrations by different mechanisms [27, 28]. However, in our study, we did not observe any evidence of protection by stevioside alone against palmitate-induced beta-cell death, suggesting that the improvement of beta-cell survival is likely due to combinations of Stevia components or the phytocomplex *in toto*.

Of note, ST3 showed similar beneficial actions with human islets under palmitate exposure, leading to reduced caspase 3/7 activation and reduced islet cell apoptosis. These novel findings support the consideration of Stevia extracts as potential tools to sustain beta-cell survival.

However, from our experiments, it is not possible to infer the possible therapeutic blood concentrations of Stevia leaf extracts or stevioside in the clinical setting. We are not aware of pharmacokinetics/pharmacodynamics studies evaluating such a point with leaf extracts. A previous report showed that after oral administration of 375 mg stevioside, 60–180 min postdose the circulating concentration of the compound was 0.1 µg/ml [52].

Palmitate exposure alters several aspects of beta-cell function and survival, inducing a series of detrimental effects identified with the term “lipotoxicity”. The main markers of lipotoxicity are ER stress and mitochondrial dysfunction. Palmitate triggers ER stress, affecting ER folding capacity and inducing overload of misfolded protein, and increase ROS production, causing mitochondrial dysfunction [3–5, 8, 39, 40, 43, 47, 50, 51, 53]. Of interest, our proteomic results showed that the ST3 extract regulates the expression of proteins related to ER and mitochondria. More specifically, it was observed an increased expression of proteins, such as HSPD1, PDIA3, PDIA6, HSPA8, and 9 ERP44 that facilitate protein folding by binding to nascent proteins and inhibiting the aggregation of misfolded proteins [54–59]. Furthermore, we found that ST3 exposure was associated with downregulation of some proteins, namely HSPA5, RCN1, whose expression is upregulated during ER stress conditions [60, 61]. Interestingly, PDIA6 protein expression has been observed to be affected by palmitate exposure in a recently published study [53].

In conclusion, the present study describes the phytochemical composition of different chemotypes of *Stevia rebaudiana* and demonstrates a role of Stevia extracts, but not of stevioside alone, on beta-cell survival protection under lipotoxic condition. This action does likely involve the ER and the mitochondrion. Whereas more studies are needed to fully clarify the mechanisms of action of Stevia on beta cells, our results foster additional efforts aiming to further assess the role of Stevia and its components on beta-cell turnover, to possibly contribute to the prevention and/or better treatment of type 2 diabetes.
